# The influence of networks of general trust on willingness to communicate in English for Japanese people

**DOI:** 10.1038/s41598-020-77108-9

**Published:** 2020-11-17

**Authors:** Takehiko Ito

**Affiliations:** Department of Information Networking for Innovation and Design, Toyo University, Akabanedai Campus, 1-7-11 Akabanedai, Kita-ku, Tokyo, 115-0053 Japan

**Keywords:** Psychology, Human behaviour

## Abstract

This study investigates the effect of a network of general trust on the willingness to communicate in English among Japanese people. Previous studies have shown that general trust positively affects the willingness to communicate in English for Japanese people. However, the network structure of general trust and its effects have not yet been revealed. The present study conducted a network analysis with 761 Japanese university students and 601 Japanese social survey participants, for 1362 participants total. Four variables regarding general trust positively affected the willingness to communicate in English for all participants, whereas one variable had a negative effect if each network was estimated for only university students or social survey participants. Centrality indices, such as node strength, closeness, and expected influence, revealed the centrality of several variables in the network of all participants. Bootstrapping methods showed the trustworthiness of the estimated edges and centrality indices. Contrary to the regression analysis, the network analysis can help us understand the profound effect of general trust on the willingness to communicate in a second language, which will prove useful for intervention studies.

## Introduction

Researchers have tried to identify the factors influencing attitudes toward second language communication among language learners. Many studies have shown the psychological processes shaping communication attitudes toward second languages. There have been many studies of Canadian, Chinese, Iranian, Japanese, and Korean learners of second language. Researchers have proposed that willingness to communicate (WTC) signals a positive attitude toward language communication^1,2^. WTC in a second language is defined^2^ as “a readiness to enter into discourse at a particular time with a specific person or persons, using a L2” (p.547). Positive correlations between WTC and frequency of communication in a second language have been reported^1,3^. Research has focused on not only psychological factors, such as confidence in language communication, Big Five personality traits, attitudes, and motivation, but also contextual factors and situational factors in predicting WTC.

Targeting Korean secondary school learners, Joe, Hiver, and Al-Hoorie^4^ examined the effects of classroom social climate as the situational factors, consisting of teacher academic support, teacher emotional support, and classroom mutual respect. These factors influence L2 (English) WTC via learners’ basic psychological needs. Next, Khajavy, Ghonsooly, Hosseini, and Choi^5^, Khajavy, MacIntyre, and Barabadi^6^, and Peng and Woodrow^7^ examined the effects of classroom environment such as teacher’s support, student’s cohesiveness, and task orientation on L2 (English) WTC, targeting Iranian university students, Iranian secondary school students, and Chinese university students, respectively. Zhang, Beckmann, and Beckmann^8^ proposed categories of situational antecedents of WTC: situation cues (i.e., teacher, class, peers, activity, and topic), situation characteristics (i.e., support, cooperation, and objectives), and the underlying dimensions of situation characteristics (i.e., negativity, positivity, and duty).

The ecological approach to research in language classrooms has recently been a focus of L2 researchers^7^. Previous studies suggest that the factors related to interpersonal trust affect L2 (English) WTC because teacher support refers to the teacher’s help, friendship, trust, and interest shown in students, while student cohesiveness means that students know, help, and support each other^9^. Even though previous studies have focused on the interpersonal relationship between teachers and students, the present study focused on general interpersonal relationships (relationships with people in general).

The present study focused on the degree of general trust, which is a feature of Japan’s social structure. According to Yamagishi^10^, interpersonal relationships and networks are less flexible in Japan than in American society. Japanese people have fewer opportunities to build new relationships, and they tend not to trust others in general. Those lower in trust limit their opportunities by interacting with smaller networks of people^11^. General trust is an individual trait, disposition, or cognitive bias toward the goodwill of others^12^. Based on this perspective, general trust is assumed to be positively associated with WTC in English because general trust is crucial in building one’s social network. Furthermore, the tendency of lower general trust among Japanese people would cause a tendency for lower WTC in English. Ito^13^ performed a regression analysis that found that general trust positively influenced WTC in English for Japanese people.

However, previous studies have not shown the network structure of general trust and its effect on WTC. There is a gap in the literature on how the variables interact and the size of the effect that each exerts. This study conducted a network analysis to examine the network structure of general trust. Network analysis allows multiple interacting factors to be considered^14^. In psychiatry, the network approach has gained popularity. The position of the nodes in the network is generated by an algorithm that makes a strongly correlated symptom cluster in the middle; symptoms with weaker connections to other symptoms go on the periphery^15^. Analyzing symptoms as a network makes it possible to examine how symptoms associate and interact with each other^16^.

By applying regression analysis, we could determine the effect (coefficient) of each variable of general trust on WTC in English, and the correlations between variables. However, such an analysis never identifies which variable of general trust serves as central (hub) between variables in relation to WTC in English. If we knew the centrality, we could understand which variable is most important for enhancing WTC in English and each variable of general trust. In other words, if the variable were removed, the effects of the other variables on WTC would be weaker. With such an analysis, we can see the effects visually; therefore, it is useful for interventions such as language education.

Latent variable models show shared variance among symptoms, whereas network analyses estimate unique variance between symptoms. Latent variable models can only show that an underlying common factor causes multiple symptoms, accounting for symptom covariation, while network models can suggest causal relationships among symptoms^17^. Furthermore, compared with structural equation modeling (SEM) and mediation analysis (MA), network analysis can use a greater number of variables at once. SEM and MA are more appropriate for a smaller number of variables, especially if there is a clear theoretical expectation for the relationships between the variables^18^.

To examine the generalization of the psychological network, university students and social survey individuals were chosen as participants. As stated in the method section, the language experiences were different between groups. If the psychological network was different between the two groups, the language experience would have effects on it.

## Methods

### Participants

The participants of the university students were 761 Japanese undergraduate students from six universities in Tokyo, Kanagawa, and Akita Prefectures (372 men, 384 women, 5 others; mean age = 19.37 years, *SD* = 1.20). Each student takes an English speaking and listening class and an English reading and writing class in the first year of study. These compulsory courses are taught by a native English speaker or a Japanese speaker of English. The participants of the social survey were 601 Japanese people living in Tokyo (284 men, 317 women; mean age = 39.65 years, *SD* = 11.00). The total number of participants was 1362. The Research Committee of the Center for English as a Lingua Franca at Tamagawa University approved this study. All methods were carried out in accordance with relevant guidelines and regulations.

The language experiences were different between the groups. “Chance of Communicating with English Speakers” was measured. The participants answered the following question, “In daily life, how often do you talk with English speakers?” Answers were 1 = *Not at All*, 2 = *Once a Month*, 3 = *Once a Week*, 4 = *Three Times a Week*, and 5 = *Every Day*. These scores were then dummy-coded 0 (*No; No chance*) for Answer 1 and 1 (*Yes; Some chances*) for Answers 2–5. It was found that the chance of social survey individuals was higher than that of university students (*χ*^2(1)^ = 15.68, *p* < 0.001). Furthermore, “Experiences in Foreign Countries” was measured. The participants answered the following question, “How long have you stayed in a foreign country?” Answers were 1 = *Never*, 2 = *Stayed for Less than Two Weeks*, 3 = *Stayed for Two Weeks to Three Months*, 4 = *Stayed for Three Months to Six Months*, and 5 = *Stayed for Over Six Months*. These scores were then dummy-coded 0 (*No; No experience*) for Answer 1 and 1 (*Yes; Some experience*) for Answers 2–5. The experience of social survey individuals was found to be higher than that of university students (*χ*^2(1)^ = 10.79, p < 0.01). To examine the generalization of the psychological network, it is important to focus on different groups who had different language experiences.

They were not asked whether they had any opportunity to communicate in English outside the language classroom, but instead were asked how often they communicated in English outside the language classroom (e.g. with customers, coworkers and friends) within a month. Of the university students, 120 participants (out of 761) communicated in English outside the language classroom within one month, while of the social survey individuals, 271 participants (out of 601) did so. In addition, they were asked whether they had any opportunities to stay in foreign countries. Of the university students, 363 participants (out of 731) had the opportunity to stay in foreign countries, while of social survey individuals, 353 participants (out of 601) did so. From these facts, not all participants would have had the opportunity to communicate in English outside the language classroom.

### Procedure

For the university students, questionnaires with scales assessing general trust and WTC in English were administered in the classes. The instructors distributed paper-based questionnaires or links to web-based questionnaires for students to complete. The first page of the questionnaire stated in Japanese that the students’ participation was voluntary and anonymous. The students provided their informed consent to participate.

For the participants of the social survey, the survey was conducted online using a web-based survey tool called Fastask. The survey company “Just System” sent emails with links to the online system of questionnaires with scales assessing general trust and WTC in English to the Japanese people enrolled. The participants could access these links on their personal computers at their own convenience. The questionnaires were the same as those of the university students. The first page of the questionnaire contained information written in Japanese that participation was voluntary and anonymous. The participants provided their informed consent to participate. After the completion of the survey, the participants received compensation.

### Questionnaire

#### General trust

The General Trust Scale was published by Yamagishi and Yamagishi^12^, and the Japanese version was used^10^. Six items were used to assess participants’ general trust (*α* = 0.86 for all participants; *α* = 0.84 for university students; *α* = 0.89 for social survey). The items are as follows: “1. Most people are trustworthy”; “2. Most people will respond in kind when they are trusted by others”; “3. Most people are trustful of others”; “4. Most people are basically honest”; “5. I am trustful”; and “6. Most people are basically good and kind.” The response options for all statements ranged from 1 (*strongly disagree*) to 5 (*strongly agree*).

#### WTC in English

The WTC in English scale was published by McCroskey^19^, and the Japanese version^3^ was used in the present study. Hashimoto^20^ and Yashima et al.^3^ used the WTC scale^19^ to measure English (L2) WTC for Japanese people who learn English as the foreign language. Since then, it has been shown repeatedly that the score on the scale is positively related to frequency of communication in English for Japanese people, and most strongly related to confidence or perceived communication competence in English (strong predictors for WTC). Even these days, many papers such as Sugawara^21^ and Yashima, MacIntyre, and Ikeda^22^ have used the Japanese version of McCroskey’s scale to measure English (L2) WTC for Japanese people who learn English as a foreign language.

The participants answered how willing they were to perform what each item described. This scale consisted of four communication contexts (talking in dyads, small groups, large meetings, and in front of an audience) with three types of receivers: strangers, acquaintances, and friends. Twelve items were used for WTC in English (*α* = 0.96 for all participants; *α* = 0.95 for university students; *α* = 0.97 for social survey). Example items are “Talking with a small group of acquaintances,” “Presenting a talk to a group of acquaintances,” “Talking with a stranger,” and “Talking in a large meeting of friends.” All items are shown in Supplementary Table S1. The response options for all statements ranged from 1 (*not at all*) to 5 (*very*).

## Results

**Figure 1:**
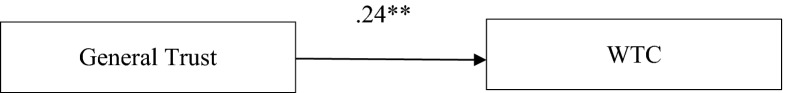
The regression analysis result of general trust on WTC in English for all participants. **p < 0.01.

Before conducting the network analysis, it was examined whether general trust positively influenced WTC in English. The regression analysis of general trust on WTC in English for all participants revealed a significant positive effect (β = 0.24, *p* < 0.01; Fig. 1), replicating the results of Ito^11^. The effect for university students was β = 0.18, *p* < 0.01, and for the social survey was β = 0.31, *p* < 0.01.

To show the network structure of general trust and its effect on WTC in English, LASSO (least absolute shrinkage and selection operator) regularization with EBIC (minimizing the extended Bayesian information criterion) model selection was used. The LASSO shrinks partial correlation coefficients to estimate a network structure whose edges show the values of the associations between nodes after controlling for the influence of all other nodes in the network, and sets small coefficients to zero^23^. Therefore, LASSO regularization with EBIC does not estimate edges that are not in the true network, and estimates edges that are in the true network based on the true network structure and sample size^23^. LASSO needs considerable power to obtain stable parameter estimates^23^.

**Figure 2:**
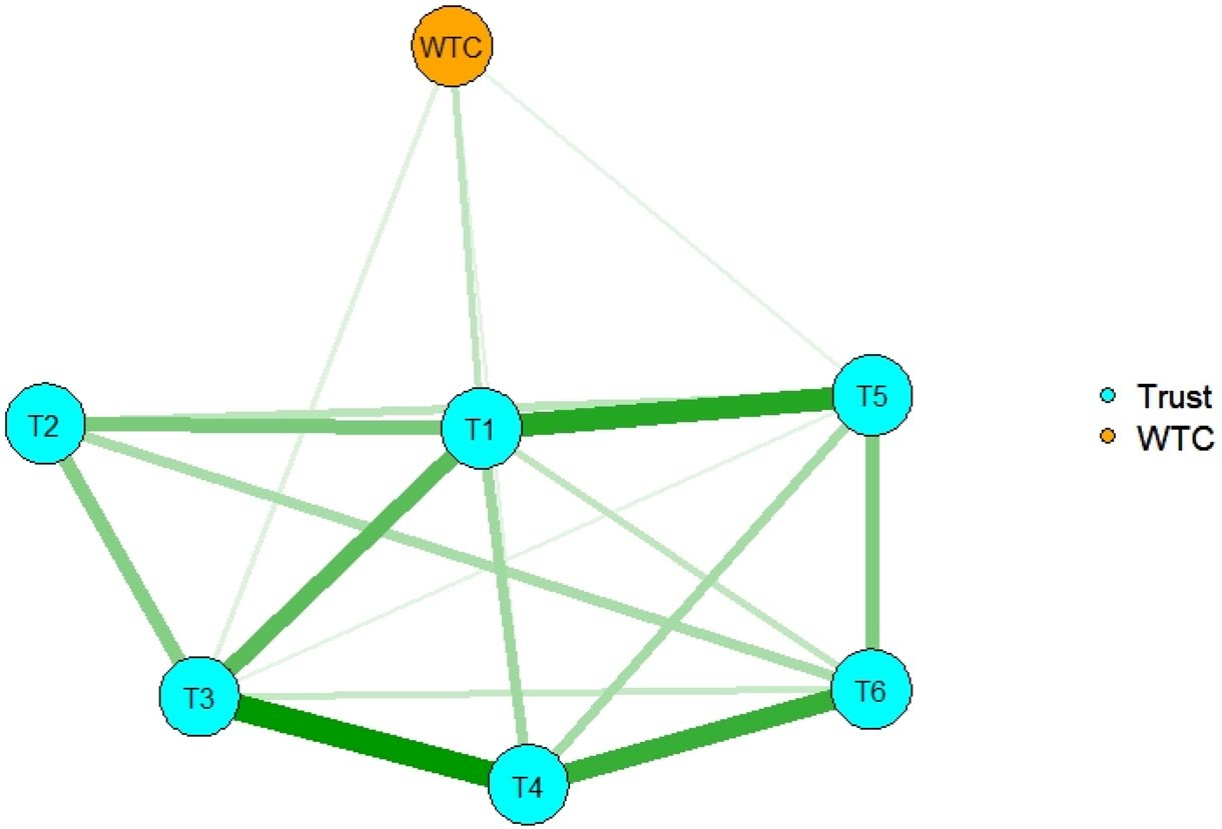
The network structure of general trust and WTC in English for all participants. Nodes represent observed variables, and links represent partial correlations between two variables after controlling for the influence of all other variables. The number in each node indicates the number of the variable in the Method section.

The R packages *qgraph*^24^ and *glasso*^25^ were used to estimate a partial correlation network for all participants using EBIC selection (Fig. 2). Four variables of general trust directly affected WTC in English: Variables 1, “Most people are trustworthy,” 3, “Most people are trustful of others,” 4, “Most people are basically honest,” and 5, “I am trustful” had positive effects. Variables 2 and 6 had indirect effects on WTC through other variables.

**Figure 3:**
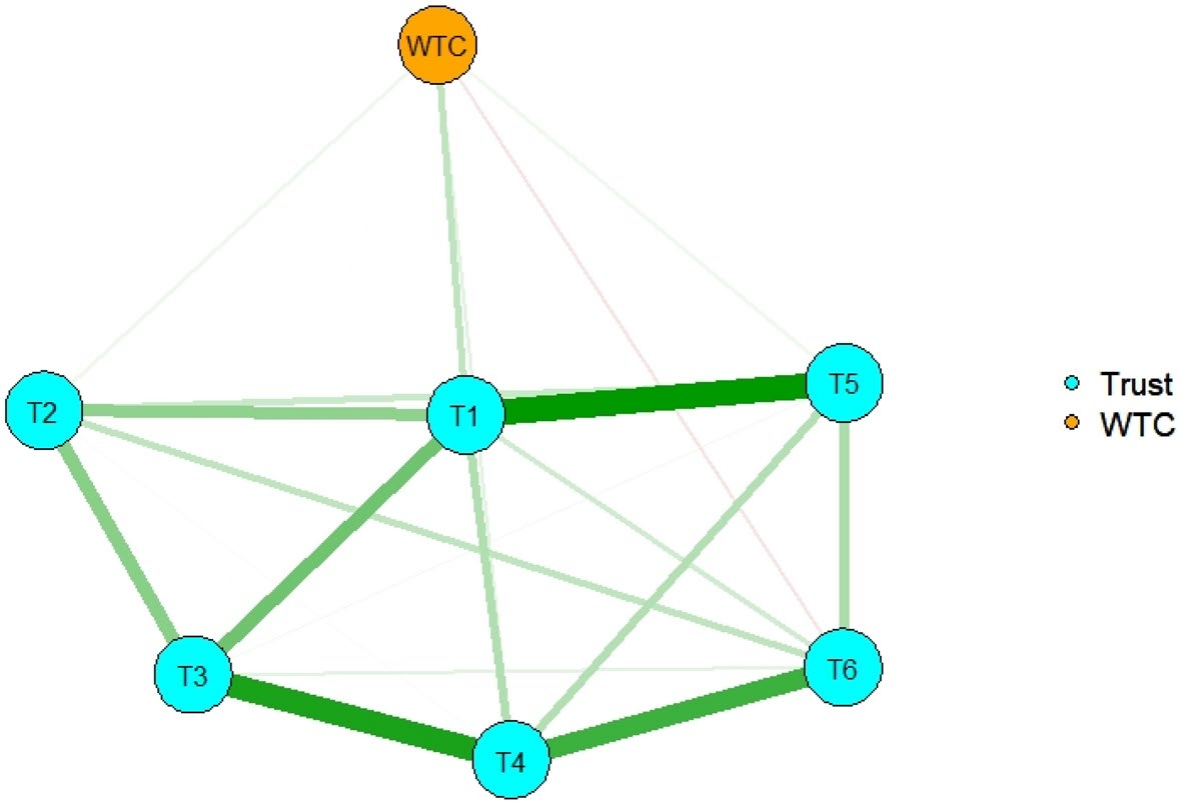
The network structure of general trust and WTC in English for university students. Nodes represent observed variables and links represent partial correlations between two variables after controlling for the influence of all other variables. The number in each node indicates the number of the variable in the Method section.

**Figure 4:**
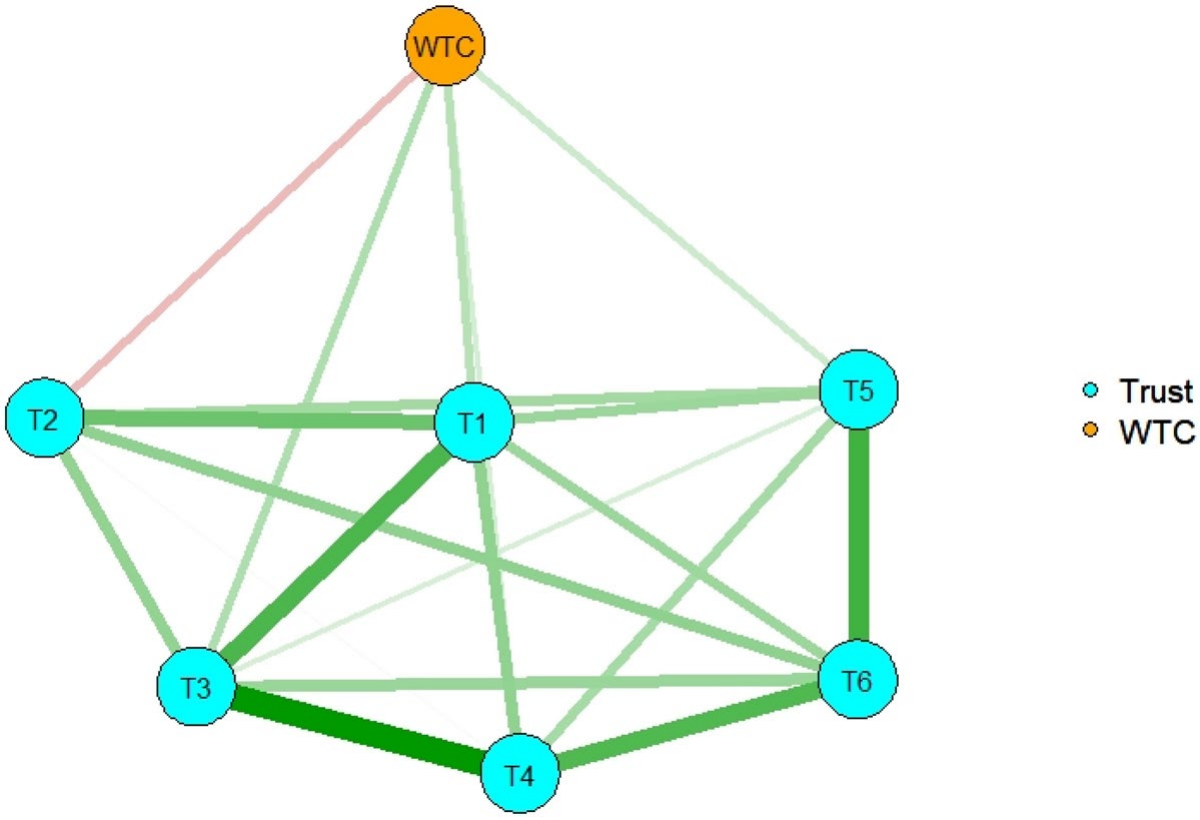
The network structure of general trust and WTC in English for the social survey. Nodes represent observed variables and links represent partial correlations between two variables after controlling for the influence of all other variables. The number in each node indicates the number of the variable in the Method section.

The R package *dplyr*^26^ was also used to estimate each group’s network structure. The correlation matrices were computed separately for university students and social surveys, and simultaneous estimation of two networks was performed: one for university students and one for the social survey, with reference to the codes of an earlier study^27^. For the network structure of university students, variables 1, 2, “Most people will respond in kind when they are trusted by others,” 4, and 5 had positive effects on WTC, which were almost the same as those of all participants; variable 6, “Most people are basically good and kind” had a negative effect on WTC (Fig. 3). For the network structure of the social survey, as with all participants, variables 1, 3, 4, and 5 had positive effects on WTC, but variable 2 had a negative effect on WTC (Fig. 4).

The importance of each node in the network is indicated by node centrality. Node strength shows the sum of the absolute partial correlation coefficients between the node and all other nodes. Closeness indicates the inverse of the sum of all the shortest paths between the node and all other nodes in the network. Betweenness refers to the number of shortest paths between two nodes passing through the node^23^. Expected influence computes node strength without taking the absolute value of edge weights^28^. The clustering coefficient is the Zhang signed clustering coefficient^29^. It encodes the tendency of a node’s neighbors to be directly connected to each other^30^. If a node’s neighbors can affect each other directly, removing a node with a very high clustering coefficient will not have a strong effect on its neighbors^27^.

**Figure 5:**
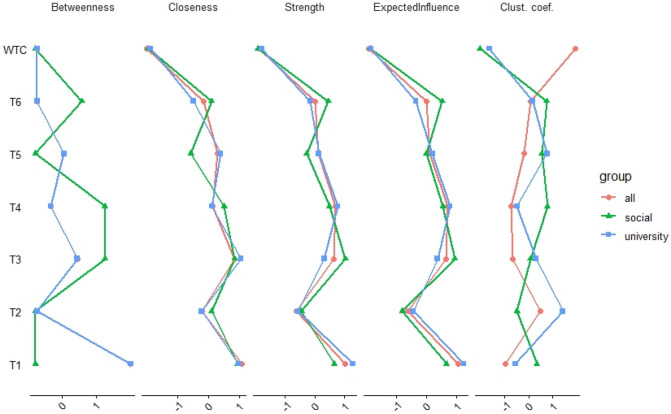
Centrality indices are shown as standardized *z*-scores. The red lines “all” indicate the values for all participants, the green lines “social” the values for social survey, and the blue line “university” the values for university students. R version 4.0.2 (https://www.r-project.org/).

Function Centrality Plot was used to estimate the centrality of the partial correlation network using EBIC selection (Fig. 5)^31^. Strength, closeness, and expected influences looked similar across the type of participants (all participants, university students, and social survey), and variables 1, 3, and 4 had strong values for the three indices. For betweenness, the total number of participants and university students had almost the same tendencies, but the social survey was different. For the clustering coefficient, each type of participant had a different tendency.

For replication concern, bootstrapping methods were used. The *bootnet* package can estimate different network models and assess the accuracy of the estimated network structure^23^. The package includes bootstrapping methods and the *CS*-coefficient. After estimating nonparametric bootstraps, *bootnet* returns plots that show the bootstrapped CIs of edge weights^23^.

**Figure 6:**
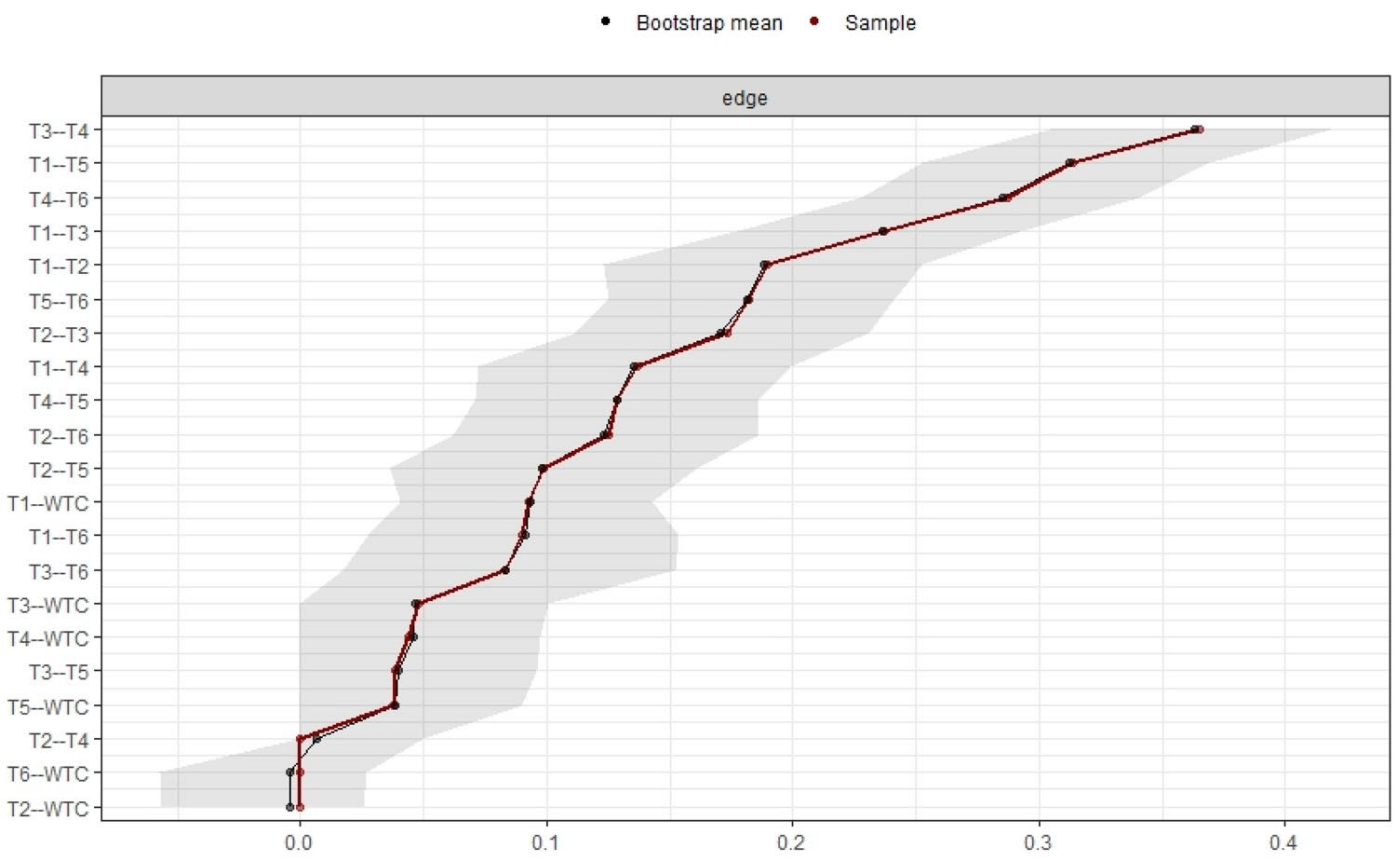
Bootstrapped confidence intervals of estimated edge-weights for the estimated network of all participants. The red line shows the sample values, and the gray area shows the bootstrapped CIs. Each horizontal line represents one edge of the network. The top is the highest edge-weight, and the bottom is the lowest edge-weight.

Figure 6 depicts bootstrapped CIs around the estimated edge weights for all participants, indicating that many edge weights likely did not differ significantly from one another. The bootstrapped CIs imply that interpreting the order of most edges in the network should be done with care^23^. In the *bootnet* function, the *nBoots* (*n* = 2500, this time) argument was used to achieve smoother plots. The *nCores* (*n* = 8, this time) argument was used to speed up bootstrapping and to use multiple computer cores. The *plot* function was used to plot bootstrapped CIs for estimates. According to the plot, the edge between variables 3 and 4 had the highest edge-weight, which had positive effects on WTC in the network of all participants. The plot also showed that the edge weight between variable 1 and WTC was the highest for the WTC effect.

**Figure 7:**
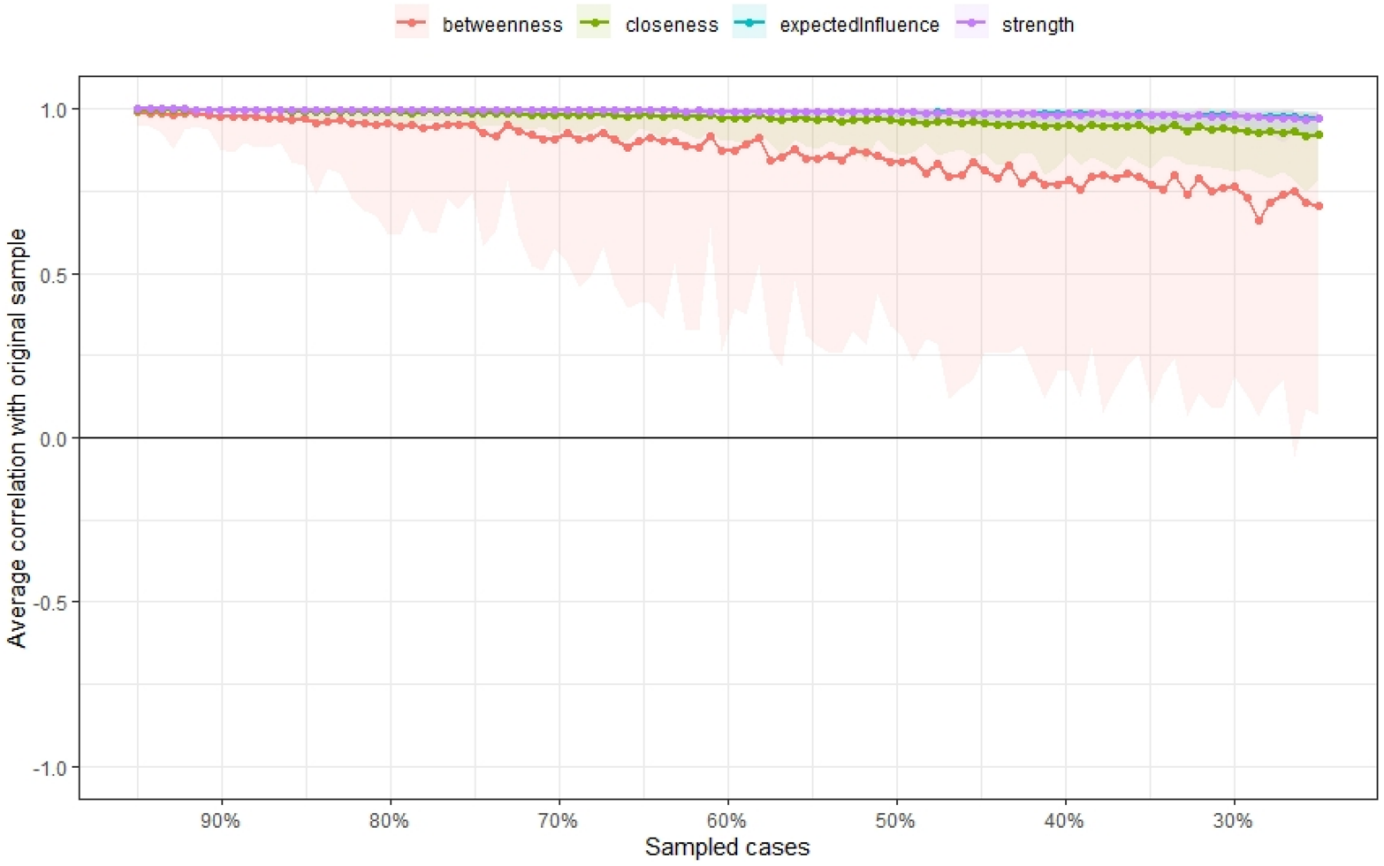
Average correlations between the centrality indices of networks sampled with participants dropped and the original participants. Colored lines depict the means, and areas depict the range from the 2.5th quantile to the 97.5th quantile.

Next, the stability of the centrality indices of the estimated network was examined based on subsets of the data for all participants. Case-dropping bootstrapping by the *corStability* function was used. Figure 7 shows the resulting plot: The stability of betweenness decreased steeply, while the stability of node strength, closeness, and expected influence improved. The *CS*-coefficient calculates the maximum proportion of cases that can be dropped to retain a correlation with the original centrality higher than (by default) 0.7^23^ The *CS coefficient* of betweenness was not good (*CS*(cor = 0.7) = 0.33). Node strength performed better (*CS*(cor = 0.7) = 0.75), as did closeness (*CS*(cor = 0.7) = 0.75) and expected influence (*CS*(cor = 0.7) = 0.75). Therefore, the index betweenness was not reliable, whereas the other three indices were reliable.

## Discussion

Before conducting network analysis, the regression analysis of general trust on WTC in English revealed a significant positive effect across the type of participants (all participants, university students, and social survey), reproducing the findings of Ito^13^.

To examine the network structure of general trust and its effect on WTC, LASSO regularization with EBIC model selection was used. For all participants, four variables directly affected WTC in English: Variables 1, 3, 4, and 5 had positive effects. Variables 2 and 6 had indirect effects on WTC through other variables. The analysis revealed how each variable interacted and influenced the WTC.

The network structure of each group was also estimated. For university students, the variables that had a positive effect on WTC in English were almost the same as those of all participants, but variable 6 had a negative effect on WTC in English. For the social survey, the variables that had a positive effect on WTC in English were the same as those of all participants, but variable 2 had a negative effect on WTC in English. Variable 2, “Most people will respond in kind when they are trusted by others,” had a positive effect on WTC in English in university students, but a negative effect in the social survey respondents. This suggests that the variable operates differently in the situations of communication in English depending on the group. In short, the reciprocal idea of general trust would lead to WTC in English among university students, but reduce WTC in English among the social survey individuals. The social survey individuals had more chances of communicating with English speakers in daily life and experiences in foreign countries than university students in the present study had. They might thus have had negative experiences in English communication, and they would expect the reciprocal idea led to negative results for communication.

The importance of each node in the network is indicated by node centrality. Strength, closeness, and expected influence looked similar across the type of participants (all participants, university students, and social survey), and variables 1, 3, and 4 had strong values, which also had a positive effect on WTC in the network of all participants. Therefore, these variables had central functions that led to WTC. In addition, the network structures of university students and social surveys were not very different because of similar tendencies in the three indices. For betweenness, the total number of participants and university students had almost the same tendencies, but the social survey was different. After using bootstrapping methods, the stability of betweenness was not high, indicating that the index was not reliable. For the clustering coefficient, each had different tendencies.

For replication concern, bootstrapping methods were used. Bootstrapped CIs around the estimated edge weights showed that many edge weights likely did not differ significantly from one another. According to the plot, the edge between variables 3 and 4 had the highest edge weight, which had positive effects on WTC in the network of all participants. Furthermore, the edge weight between variable 1 and WTC was the highest for the WTC effect. Variable 1 had a positive effect on WTC in the estimated network of all participants, and values of strength, closeness, and expected influence were high; therefore, variable 1 had a large impact on WTC in English.

The stability of the centrality indices of the estimated network was examined based on subsets of the data of all participants. The stability of betweenness decreased steeply, while the stability of node strength, closeness, and expected influence improved. The *CS coefficient* of betweenness was not good, whereas node strength, closeness, and expected influence performed better. Therefore, the index betweenness was not reliable, while the indices of node strength, closeness, and expected influence were reliable.

Cumulative theoretical knowledge about social psychology has shown that general trust influences human perception, affection, and behavior. However, the network structure of general trust and its effect on human attitudes have not been reported. In this study, network analysis showed that several variables that made up a network themselves positively influenced WTC in English. If each group’s network was estimated, one variable negatively influenced WTC, which regression analysis could not determine. Several variables that had positive effects on WTC also showed high centralities in the network structure. Network analysis revealed the central variables to work for the WTC.

Contrary to the regression analysis, network analysis examined which variable of general trust functions as central (hub) between the variables in relation to WTC in English. If we knew the centrality, we could determine which variable is most important to enhance WTC in English and each variable of general trust. In other words, if the variable were removed, the effects of the other variables on WTC would be weaker. With this analysis, we can see the effects visually; therefore, it is useful for interventions such as language education. According to the result of centrality indices for university students, Variable 1 “Most people are trustworthy” had the highest strength and a strong positive connection with WTC in English. This means that the variable was a hub in the network structure. In the language classrooms, if teachers enhance this variable, it activates other variables of general trust and WTC in English among university students. The results suggest how to enhance learner’s positive attitudes toward English communication.

Previous studies suggest that the factors related to interpersonal trust are influential on L2 (English) WTC. Even though these studies focused on the interpersonal relationship between teachers and students, the present study focused on general interpersonal relationships (relationships with people in general). In this study, general trust, which reflects features of the social structure, was examined. According to Yamagishi^10^, Japanese people have fewer opportunities to build new relationships and they tend not to trust others in general. General trust has the power to maximize profitable interpersonal relations, and the present study reveals the positive relationships between general trust and WTC in English. This implies that the lower tendency of general trust in Japanese society would cause a tendency for lower WTC in a second language.

However, a limitation is that this study did not focus on other nationalities, such as the American population. According to Yamagishi^10^, interpersonal relationships and networks are flexible in Western countries such as America. Individuals have opportunities to build new relationships and tend to trust others in general. Based on these findings, we expect the general trust of a western population to be high, and its network structure and effects on WTC in the second language to differ from those found in the present study. To generalize this study, future studies should focus on the general trust of Western societies.

## Conclusion

The present study showed the network structure of general trust and its effect on WTC in English for Japanese people. Contrary to the regression analysis, the findings from network analysis showed how each variable interacted and which variable was central in its effects on WTC. These findings can help us understand the profound effect of general trust on WTC in a second language. These results can also help us prepare intervention studies by showing specific variables that directly affect WTC in a second language and how they interact with each other.

## Supplementary information

Supplementary information.

## Data Availability

The datasets generated during the current study are available from the corresponding author on reasonable request.
