# Supplementary material for: The influence of networks of general trust on willingness to communicate in English for Japanese people
Source: Sci Rep. 2020 Nov 17;10:19939. doi: 10.1038/s41598-020-77108-9 (PMC7672104; doi:10.1038/s41598-020-77108-9)
Supplement: Supplementary file 1 — Supplementary information. [file 41598_2020_77108_MOESM1_ESM.docx]

The influence of networks of general trust on willingness to communicate in English for Japanese people

Takehiko Ito ^a^

^a^ Department of Information Networking for Innovation and Design, Toyo University

Akabanedai Campus, 1-7-11 Akabanedai, Kita-ku, Tokyo, Japan, 115-0053

Correspondence:

Takehiko Ito

Tel: 090-4968-2915

E-mail: ito9041@toyo.jp

Akabanedai Campus, 1-7-11 Akabanedai, Kita-ku, Tokyo, Japan, 115-0053

Supplementary Table S1 The items of WTC in English. This scale consisted of four communication contexts (talking in dyads, small groups, large meetings, and in front of an audience) with three types of receivers: strangers, acquaintances, and friends.

Strangers:

Talking with a stranger

Talking with a small group of strangers

Talking in a large meeting of strangers

Presenting a talk to a group of strangers

Acquaintances:

Talking with an acquaintance

Talking with a small group of acquaintances

Talking in a large meeting of acquaintances

Presenting a talk to a group of acquaintances

Friends:

Talking with a friend

Talking with a small group of friends

Talking in a large meeting of friends

Presenting a talk to a group of friends
